# The Immunomodulatory Mechanisms of BTK Inhibition in CLL and Beyond

**DOI:** 10.3390/cancers16213574

**Published:** 2024-10-23

**Authors:** Qu Jiang, Yayi Peng, Carmen Diana Herling, Marco Herling

**Affiliations:** 1 Department for Hematology, Cell Therapy, Hemostaseology, and Infectious Diseases, University Hospital of Leipzig, 04103 Leipzig, Germany; yayi.peng@medizin.uni-leipzig.de (Y.P.); carmen.herling@medizin.uni-leipzig.de (C.D.H.); marco.herling@medizin.uni-leipzig.de (M.H.); 2 Cancer Center Central Germany (CCCG), Leipzig-Jena, 04103 Leipzig, Germany

**Keywords:** CLL, BCR, BTK inhibition, immunomodulatory mechanisms, immune cell micromilieu

## Abstract

Bruton’s tyrosine kinase (BTK), a cytoplasmic tyrosine kinase, plays a pivotal role in B cell biology and function. As an essential component of the B cell receptor (BCR) signaling pathway, BTK is expressed not only in B cells but also in myeloid cells, including monocytes/macrophages, dendritic cells, neutrophils, and mast cells. BTK inhibitors (BTKis) have revolutionized the treatment of chronic lymphocytic leukemia (CLL) and other B cell malignancies. Besides their well-characterized role in inhibiting BCR signaling, BTKis also exert significant immunological influences outside the tumor cell that extend their therapeutic potential and impact on the immune system in different ways. This work elucidates the immunomodulatory mechanisms associated with BTK inhibition, focusing on CLL and other clinical contexts. We discuss how BTK inhibition affects various immune cells, including B cells, T cells, and macrophages. The effects of BTKis on the profiles of cytokines, also fundamental parts of the tumor microenvironment (TME), are summarized here as well. This review also appraises the implications of these immunomodulatory actions in the management of autoimmune diseases and infections. Summarizing the dual role of BTK inhibition in modulating malignant lymphocyte and immune cell functions, this paper highlights the broader potential clinical use of compounds targeting BTK.

## Simple Summary

Inhibitors of Bruton’s tyrosine kinase (BTK) have changed how we treat chronic lymphocytic leukemia (CLL), a B cell malignancy. These inhibitors are known for blocking signals in B cells that help cancers derived from these immune cells grow, but they also have important effects on the non-malignant immune system. This work explains how BTK inhibitors influence different components of the immune system, such as B cells, T cells, and macrophages. It also reviews their impact on the generation of immune-signaling molecules or their effect on the tumor microenvironment. We also explored how these effects might be useful in treating autoimmune diseases and infections. By understanding how BTK inhibitors can impact cancer cells and the immune system, this paper highlights the potential for these drugs to be used in a wider range of medical conditions.

## 1. Introduction

**Figure 1:**
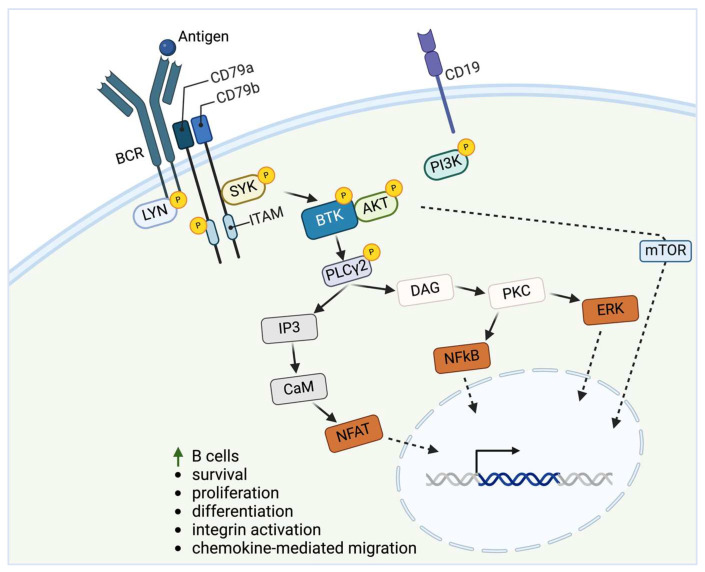
B cell receptor signaling pathway [5]. Abbreviations: BCR = B cell receptor; SYK = spleen tyrosine kinase; LYN = Lyn kinase; ITAM = immuno-receptor tyrosine-based activation motif; BTK = Bruton’s tyrosine kinase; PI3K = phosphoinositide 3-kinase; AKT = protein kinase B; PLCγ2 = phospholipase C gamma 2; P = phosphorylation; IP3 = inositol 1,4,5-trisphosphate; CaM = calmodulin; NFAT = nuclear factor of activated T cells; DAG = diacylglycerol; PKC = protein kinase C; NFκB = nuclear factor kappa-light-chain-enhancer of activated B cells; ERK = extracellular signal-regulated kinase; mTOR = mechanistic target of rapamycin. Image created in BioRender.com (accessed date 1 Oct. 2024). A list of abbreviations is included in Supplementary Table S1.

Bruton’s tyrosine kinase (BTK) is a key mediator in the B cell receptor (BCR) signaling pathway and can be targeted by BTK inhibitors (BTKis). The apical BCR complex includes an immunoglobulin transmembrane protein linked to two signal-transmitting subunits, CD79a and CD79b [1,2]. Upon engagement, CD79a and CD79b are co-recruited, leading to the activation of spleen tyrosine kinase (SYK) and LYN kinase from Src family of tyrosine kinases. These in turn phosphorylate immuno-receptor tyrosine-based activation motifs (ITAMs), triggering a cascade that activates BTK and phosphoinositide 3-kinase (PI3K) [3,4]. Upon activation, BTK phosphorylates and activates phospholipase C gamma 2 (PLCγ2), initiating a positive feedback loop [5]. PLCγ2 can regulate downstream mediators such as the mitogen-activated protein kinase (MAPK) family, including extracellular signal-regulated kinases 1 and 2 (ERK1/ERK2) and activate the transcription of the nuclear factor of activated T cells (NFAT). Autoantigens have been shown to drive BCR dependent activation of the nuclear factor kappa-light-chain-enhancer of activated B cells (NFκB) through a cascade that includes SYK, BTK, and protein kinase Cβ (PKCβ) [6]. This cascade eventually activates various cellular processes including integrin activation, chemokine-mediated migration, as well as the differentiation, survival, and proliferation of B cells (Figure 1).

BTKis have emerged as breakthrough therapies in the treatment of B cell malignancies, particularly in chronic lymphocytic leukemia (CLL) [7]. In CLL, malignant B cells benefit from BTK-mediated pro-survival signals that are triggered by tonic patterns of BCR engagement, e.g., constantly (auto)antigen-driven or autonomously by BCR autoreactivity [8]. A functional BCR has been centrally implicated in both the development and persistence of CLL [9,10,11,12]. Other B cell lymphomas also have implemented chronic active BCR signaling in their pathogenesis [13]. Ibrutinib was the first BTKi approved for the treatment of CLL in 2014 [14] and there are over 20 BTKis in development (Table 1). Several BTKis have been approved for clinical use. These include irreversible BTKis such as acalabrutinib, zanubrutinib, and tirabrutinib, which covalently bind to BTK, similar to ibrutinib, but exhibit higher target selectivity, and reversible BTKi pirtobrutinib [7,15].

For the longest time, our mechanistic concept of BTKis relied on blocking such chronic active BCR signal transmission in the malignant B cell, leading to the altered migration and apoptosis of the neoplastic clone [16]. However, recent studies revealed that BTKis also have significant modulatory effects on the non-malignant cells of the immune system. These effects may contribute to cellular and clinical outcomes, including toxicity, associated with inhibiting BTK function [17,18,19,20]. For instance, BTK is also expressed in monocytes/macrophages, dendritic cells (DCs), neutrophils, and mast cells (MCs) [21,22,23,24]. BTK inhibition, hence, can alter the immune environment, leading to reduced inflammation or suppressed (auto)immune reactions [25]. This in turn may cause CLL patients to experience immune-related side effects, such as an increased risk of infection [26]. On the other hand, ibrutinib has been approved for the treatment of chronic graft-versus-host disease (cGvHD) [27]. Furthermore, long-term BTKi therapy may contribute to the reconstitution of immune function in CLL patients [28]. BTKis might also have protective effects against the damages by coronavirus disease 2019 (COVID-19) in patients with CLL [29].

The immunomodulatory properties of BTK have prompted applications of BTKis beyond the field of oncology. There is growing interest in using these inhibitors to treat autoimmune diseases in which dysregulated B cell activity and chronic inflammation play a key role [30,31]. Additionally, BTK inhibition can significantly impact the balance and homeostasis of various immune cell populations, including T cells, B cells, and macrophages, underscoring its importance for different immune functions, including responses to infections [19]. A better understanding of the multifunctionality of BTKis is crucial for optimizing their use in cancer as well as in infectious and immune diseases.

## 2. The Role of BTK in Immune Regulation

The role of BTK in BCR signaling was first studied in X-linked agammaglobulinemia (XLA), a B cell immunodeficiency characterized by an almost complete block of B cell development at the pre-B cell stage [32,33,34,35]. In B cells, BTK supports development, maturation, survival, proliferation, and antibody production through its role as a downstream kinase in BCR signaling [32,33,36]. BTK is also involved in many other signaling pathways in B cells, including the chemokine receptor, the Toll-like receptor (TLR), and Fc receptor signaling [6].

**Figure 2:**
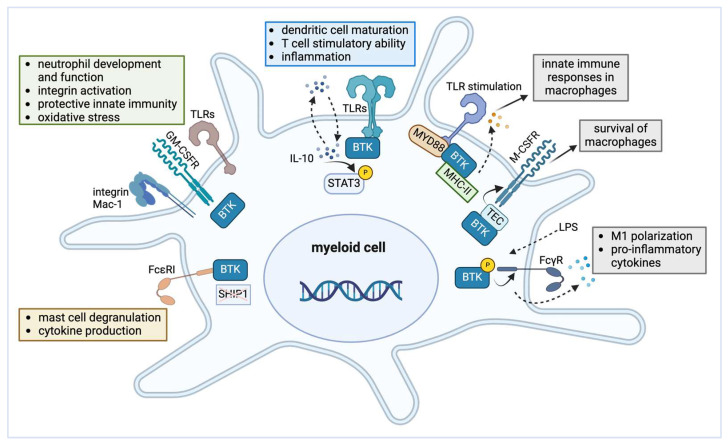
The role of BTK in myeloid cells; details are in the main text. (I) BTK in neutrophils (green box); (II) BTK in conventional dendritic cells (blue box); (III) BTK in macrophages (gray box); (IV) BTK in mast cells (orange box). Abbreviations: BTK = Bruton’s tyrosine kinase; TLR = Toll-like receptor; GM-CSFR = granulocyte–macrophage colony-stimulating factor receptor; FcεRI = high-affinity IgE receptor; SHIP1 = SH2-containing inositol phosphatase 1; IL-10 = interleukin-10; STAT3 = signal transducer and activator of transcription 3; P = phosphorylation; MYD88 = myeloid differentiation primary response 88; MHC-II = major histocompatibility complex class II; TEC = tyrosine kinase expressed in hepatocellular carcinoma; LPS = lipopolysaccharide; FcγR = Fc gamma receptor; pink X = deficient. Image created in BioRender.com (access date 1 October 2024).

The expression of BTK is not restricted to B cells (Figure 2). BTK has been shown to be important in neutrophils. In a mouse model of XLA, it was demonstrated that neutrophil development and function critically depended on BTK [37]. BTK was required for multiple integrin Mac-1 (CD11b/CD18) activation events involved in neutrophil recruitment and function during sterile inflammation [38]. A study on pneumococcal pneumonia and sepsis suggested that BTK is critical for regulating myeloid cell-mediated innate host defense, particularly in neutrophils [39]. Nadeem et al. investigated the role of BTK and its inhibition on oxidizing enzymes in DCs, neutrophils, and B cells during sepsis-induced acute kidney injury [40]. This study shed light on the involvement of BTK in inflammatory and oxidative signaling in immune cells, including neutrophils.

Melcher et al. highlighted the roles of BTK in macrophage colony-stimulating factor (M-CSF) receptor signaling pathways that influence macrophage survival [41]. Additionally, it has been demonstrated that intracellular major histocompatibility complex (MHC) class II molecules interact with BTK to maintain its activation, thereby promoting TLR-triggered innate immune responses in macrophages [42,43]. BTK activation in macrophages leads to the production of pro-inflammatory cytokines such as tumor necrosis factor-alpha (TNF-α) and interleukin-6 (IL-6), which are essential for mounting an effective immune response [44]. Gabhann et al. highlighted the importance of BTK in affecting the balance between pro-inflammatory (M1) and anti-inflammatory (M2) macrophages, thus contributing to immune homeostasis [45].

As professional antigen-presenting cells (APCs), conventional (c)DCs are involved in the initiation of a T cell immune response, thus linking the innate with the adaptive immune system. A study showed that a genetic defect in BTK (in XLA) did not affect cDC differentiation and maturation and that such XLA-cDCs can still fully function as APCs in T cell-mediated immune responses [23]. In contrast, BTK was reported as a key molecule in TLR9 signaling in plasmacytoid (p)DCs. There, it enhances cytokine production and the expression of costimulatory molecules [46]. Conversely, BTK−/− murine cDCs exhibited a more mature phenotype, stronger T cell stimulatory ability, and enhanced inflammation [47]. These roles of BTK in cDCs might be mediated by the autocrine secretion of IL-10 and subsequent activation of the signal transducer and activator of transcription 3 (Stat3), a transcription factor critical for immune tolerance [47]. Therapeutically, BTK inhibition promotes the differentiation of monocyte-lineage cDCs and enhances antitumor T cell immunity [48].

BTK has also been studied in MCs [49,50,51]. Already in 1998, it was reported that BTK-deficient MCs exhibited significant abnormalities in Fcε receptor I (FcεRI)-dependent activation and function [49]. BTK inhibition blocked FcεR cross-linking-induced degranulation in MCs [50]. Moreover, BTK was shown to play a role in FcεRI-mediated signaling and effector functions in SH2-containing inositol-5′-phosphatase 1 (SHIP1)-deficient MCs, in which the reduced activation could be reversed by pharmacologic BTK inhibition [51].

## 3. BTK Inhibition in CLL

### 3.1. Mechanisms of Action in CLL

BTK is overexpressed in CLL cells as compared to normal B cells, and its dysregulated activity marked by overexpression and constitutive phosphorylation contributes to persistent signaling, which promotes leukemic growth [16]. The treatment for CLL has significantly advanced with the emergence of BTKis as a central therapeutic strategy. Ibrutinib, the first-in-class orally bioavailable BTKi, irreversibly binds to the cysteine 481 (C481) residue of BTK, thereby blocking the phosphorylation of downstream kinases in the BCR cascade, inducing modest apoptosis in CLL cells, independent of microenvironmental survival signals [52,53]. Further studies showed that ibrutinib inhibits BCR-controlled signaling and the integrin-mediated adhesion and migration of CLL cells [54,55]. The critical role of functional BTK in the development and expansion of CLL was confirmed in a *T cell leukemia/lymphoma 1A (TCL1A)* transgenic CLL mouse model [56,57]. Therein, the inhibition of BTK activity through either targeted genetic inactivation or ibrutinib significantly delayed the development of murine CLL [58]. Vela et al. underlined a novel mechanism of action for ibrutinib, which blocks downstream signaling of the BCR, disrupting stromal microenvironment interactions and B cell cytokine signaling [59]. Besides its intended BTK effects, ibrutinib also deactivates several off-targets, including epidermal growth factor receptor (EGFR), ErbB2, interleukin-2-inducible T cell kinase (ITK), and tyrosine kinase expressed in hepatocellular carcinoma (TEC) [60]. While these off-target actions likely contribute to the anti-CLL effect, they are also associated with side effects such as atrial fibrillation, hemorrhage, or hypertension [60]. There are second-generation BTKis, such as acalabrutinib and zanubrutinib, with higher specificity to BTK and reduced off-target kinase inhibition [15].

Mechanisms of resistance to BTKis have been intensively investigated. Resistance to ibrutinib and acalabrutinib often involves mutations of the central target residue C481S [61,62]. Further studies revealed gain-of-function mutations in *PLCγ2* (R665W, L845F, S707Y), associated with a secondary ibrutinib resistance mechanism in CLL [61,63,64]. Pirtobrutinib, a reversible BTKi, was designed to treat resistant CLL caused by C481S mutations [65]. It has shown efficacy in relapsed or refractory B cell malignancies [66] and is effective against both wild-type and mutant BTK-mediated signaling in CLL [65]. Enrichment of BTK L528T mutations has been observed in CLL patients progressing under zanubrutinib. Both structural and experimental data suggest that this mutation interferes with the binding of both ATP and zanubrutinib to BTK [67]. Additionally, integrin signaling, especially very late activation antigen 4 (VLA-4, CD49d/CD29) activation, might contribute to resistance towards BTKis [68]. Consequently, a new class of BTK-targeting drugs, BTK protein degraders, has been developed for the treatment of CLL [69,70]. Studies have shown the broad activity of BTK degraders across the spectrum of wild-type and mutant BTKs, including C481S, L528W, T474I, and V416L [71]. These studies provide insights into new approaches for overcoming resistance to BTKis in CLL. Preclinical and clinical studies have demonstrated the potential of combinatorial therapy to overcome BTKis’ resistance, including combinations with other targeted therapies [7]. Current BTKis in phase II–III clinical trials for CLL are presented in Supplementary Table S2.

### 3.2. Immunomodulatory Effects in CLL

**Figure 3:**
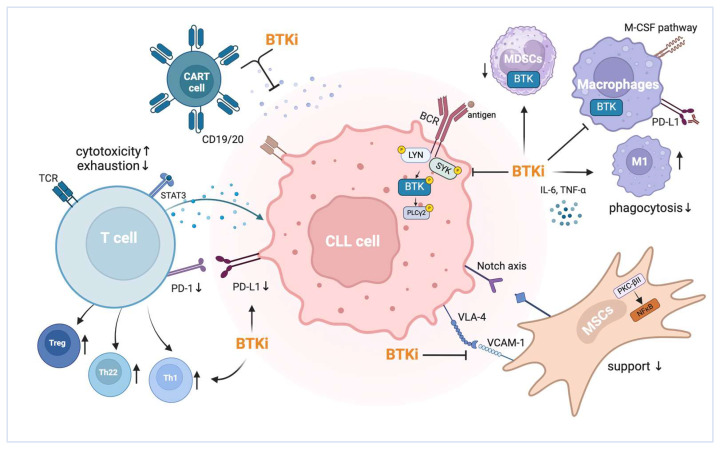
BTK inhibition in CLL cells and their environment; details are in the main text. Abbreviations: BCR = B cell receptor; BTK = Bruton’s tyrosine kinase; SYK = spleen tyrosine kinase; LYN = Lyn kinase; PLCγ2 = phospholipase C gamma 2; PKC-βII = protein kinase C β II; NFκB = nuclear factor kappa-light-chain-enhancer of activated B cells; VLA-4 = very late activation antigen 4; VCAM-1 = vascular cell adhesion molecule 1; MSC = mesenchymal stromal cells; IL-6 = interleukin-6; TNF-α = tumor necrosis factor-alpha; PD-L1 = programmed cell death ligand 1; PD-1 = programmed cell death protein 1; MDSCs = myeloid-derived suppressor cells; CAR = chimeric antigen receptor; Treg = regulatory T cells; Th = T helper. Image created in BioRender.com (access date 1 October 2024).

The effects of BTK inhibition in CLL go beyond mere reduction in canonical BCR signals in the leukemic cell. Since BTK and its family members are expressed not only in B cells, but also in other cell types, BTKis, whether highly selective or not, also affect non-leukemic cells [72]. In fact, aberrant BCR signaling in CLL drives a complex interplay between the leukemic cells and their tumor microenvironment (TME), involving cross-talks with accessory T lymphocytes, macrophages, and stromal cells (Figure 3) [73,74].

Ibrutinib can markedly increase CD4+ and CD8+ T cell numbers in CLL patients, most prominently in effector/effector memory subsets, leading to decreased Treg/CD4+ T cell ratios alongside alleviation of the tolerogenic and immunosuppressive skewing that is characteristic of CLL [75]. Mechanistically, upon ibrutinib treatment, the downregulation of programmed cell death ligand 1 (PD-L1) on CLL cells and of programmed cell death protein 1 (PD-1) on T cells is observed, potentially reverting pseudo-exhaustion and enhancing antitumor immune responses [17]. Also, ibrutinib alters CLL and T cell homing receptor expression [76]. Furthermore, BTK inhibition increases CD8+ T cell cytotoxicity and immune synapse formation [77]. The combination of ibrutinib with checkpoint blockade could further improve CD8+ T cell function, highlighting the immuno-therapeutic potential of BTK inhibition via restoring T cell composition and functionality in CLL [18]. This effect may generally be harnessed to improve CAR-T cell efficacy, as supported by preclinical studies showing improved tumor clearance when BTK inhibition is combined with anti-CD19 CAR-T cells [78]. Moreover, ibrutinib increases T cell counts and skews T cell differentiation to T helper 1 (Th1) cells in the presence of myeloid-derived suppressor cells (MDSCs) [72]. Ibrutinib also shows positive effects on IL-22+ (Th22) and FoxP3+ (Treg) cell numbers in the presence of MDSCs and monocytes [72]. However, a progressive decrease in MDSCs was observed during ibrutinib therapy, potentially due to a direct effect on BTK in MDSCs or an indirect effect mediated by reduced signaling from CLL cells that typically occurs following BCR activation [72]. The extent of this multifaceted modulation of T cell (and CLL cell) motility and adhesive properties as well as T cell cytotoxicity by ibrutinib might also vary across CLL patients, as they are likely intrinsically determined, and by that underlie the variable clinical treatment responses [76]. Additionally, the structural homology of BTK to T cell ITK suggested BTKis as viable options in T cell lymphomas. However, single-agent activities were rather low with respect to the induction of T cell apoptosis, e.g., in models of T cell prolymphocytic leukemia [79,80,81]. More selective BTKis like zanubrutinib and acalabrutinib do not impact T cell function as does ibrutinib [75,82]. In fact, significant changes in T cell counts were observed during the first 20 weeks of acalabrutinib treatment, but CD4+ and CD8+ T cell counts decreased with prolonged use [75]. Further research is needed in this area.

BTKis can also reprogram tumor-associated macrophages (TAMs) from the M2 to M1 phenotype, reducing their tumor-promoting activities and enhancing their immune-mediated clearance of CLL cells [41]. Studies have shown that macrophage depletion impairs CLL engraftment, reduces leukemic growth, and improves survival in mouse models [74,83]. Ibrutinib affects macrophages, particularly monocytic nurse-like cells (NLCs) in CLL, which function similarly to TAMs. Ibrutinib alters the phenotype and function of NLCs by diminishing their phagocytic ability and enhancing their immunosuppressive profile, as evidenced by the increased expression of M2 markers [84]. PD-L1 expression on macrophages also promotes an M2 phenotype, contributing to immune evasion, but blocking the PD-1/PD-L1 pathway with ibrutinib could reverse these dysfunctions and restore the metabolic and antitumor activity of monocytes/macrophages in CLL [85]. In that context, it is very intriguing that in some instances, ibrutinib, mainly in the context of CLL, can cause hemophagocytic lymphohistiocytosis (HLH), an often-fatal T/NK cell- and cytokine-driven hyperinflammatory syndrome [86].

Stromal cells enhance the immune-evasive phenotype of CLL cells by increasing PD-L1 expression through the Notch signaling pathway—myelocytomatosis oncogene—an enhancer of the zeste homolog 2 (Notch–c-Myc–EZH2) signaling axis and by upregulating protein kinase C (PKC)-βII, which activates NFκB, crucial for malignant B cell survival [87,88]. BTKis, targeting such CLL-intrinsic pathway activation that is elicited by milieu stimuli, disrupt downstream cascades like PI3K–protein kinase B (PI3K–AKT) and NFκB networks, potentially weakening the TME’s support for CLL cells. In turn, inhibiting stromal cell kinases such as PKCβ or LYN with BTKis has been shown to reduce stromal cell-derived pro-survival signals, thereby enhancing treatment outcomes [88,89].

Together, the immunomodulatory effects of BTKis present opportunities for combining these agents with other (immuno)therapeutic modalities, such as checkpoint inhibitors, CAR-T cells [18,75], or bispecific antibodies, to enhance antitumor immunity towards more durable remissions. While BTKis can restore immune function, they also carry a risk of immunosuppression, affecting normal B cells as well as other immune cells. This, in fact, was the rationale for BTKis in cGvHD [27,90], a multi-cellular alloreaction involving various immune cells, including B cells, T cells, stromal cells, and macrophages [91]. At this point, it is important to emphasize, when interpreting experimental data from models and clinical observations, that the effects of (lineage-specific) genetic BTK depletion are not entirely identical to the organismal impact of pharmacologic BTK inhibition by currently available small molecules. Moreover, until better discernable from the desired effects of therapeutic BTK targeting, its immunosuppressive aspect requires the careful prophylaxis of infections and monitoring in the context of BTKi treatment. It also remains to be addressed if (non)selective BTK inhibition exerts the same global effects in non-CLL B cell neoplasms such as mantle cell lymphoma, marginal zone lymphoma, Waldenström’s macroglobulinemia, and others.

## 4. Immunomodulatory Effects of BTK Inhibitors Beyond Lymphoid Neoplasms

### 4.1. Autoimmune Diseases

Since the discovery of BTK in XLA [35], studies investigating its role in autoimmune diseases have reinforced the link between BTK and autoimmune phenomena [20,92]. Given BTK’s key involvement in BCR signaling, its inhibition presents an attractive therapeutic approach for autoimmune diseases involving abnormal B cell function, such as rheumatoid arthritis (RA), systemic lupus erythematosus (SLE), or multiple sclerosis (MS). Current clinical trials testing BTKis in RA, SLE, and MS are summarized in Supplementary Table S3.

#### 4.1.1. Rheumatoid Arthritis

RA, one of the common autoimmune disorders, is a chronic inflammatory systemic and locally destructive disease. Its characteristic synovitis and joint damage are the results of complex autoimmune and inflammatory processes involving components of both the innate and adaptive immune systems [93]. As BTK plays a fundamental role in the development, differentiation, and proliferation of B cells, it is an attractive target in RA [94]. The BTKi CGI1746 has shown to block BCR-dependent B cell proliferation and in prophylactic regimens to reduce autoantibody levels, as well as to abolish FcγRIII-induced TNF-α, IL-1β, and IL-6 production in macrophages, in murine collagen-induced arthritis [44]. It also decreases intra-articular cytokine levels and ameliorates disease in myeloid- and FcγR-dependent autoantibody-induced arthritis [44]. Another potent BTKi, GDC-0834, showed an anti-arthritic effect by inhibiting BTK phosphorylation [94]. Further research by Jang et. al. presented the efficacy of poseltinib in rodent models of arthritis [95]. Poseltinib has also been shown to suppress B cell and monocyte activation and to ameliorate arthritis in mice [96]. In an adjuvant-induced arthritic rat model, an irreversible BTKi, CHMFL-BTK-11, reduced the inflammatory response by blocking the proliferation of activated B cells, inhibiting the secretion of the inflammatory factors IgG1, IgG2, IgM, IL-6, and PMΦ phagocytosis, and stimulating IL-10 secretion [97]. Furthermore, the BTKi BMS-986142 enhanced the efficacy of standard-of-care agents (e.g., methotrexate, the TNF-α antagonist etanercept, and the murine form of CTLA4-Ig) in experimental models of RA [98]. Another BTKi, fenebrutinib, suppressed B cell- and myeloid cell-mediated disease aspects in an in vivo rat model of arthritis [99]. Recently, a novel tricyclic BTKi, SOMCL-17-016, was shown to suppress B cell responses and osteoclastic bone erosion in RA [100]. Moreover, there have been several clinical trials evaluating the efficacy and safety of BTKis in RA (Supplementary Table S3). However, a published phase II study of BMS-986142 in RA showed limited efficacy [101], emphasizing future refined approaches.

#### 4.1.2. Systemic Lupus Erythematosus

SLE, as a chronic autoimmune condition, is characterized by dysregulated innate and adaptive immune responses involving B cells, T cells, DCs, and macrophages, leading to the activation of the complementary system, production of autoantibodies, cytokines, and chemokines, and formation of immune complexes [102]. BTK is considered a key enzyme in the signaling pathways of B cells and myeloid cells that are critical in the pathogenesis of SLE [103]. Experimental overexpression of BTK in B cells leads to the development of SLE-like autoimmune pathology in mice, which could be alleviated by BTKi treatment [104]. In support, the selective BTKi, RN486, suppresses glomerulonephritis in lupus-prone mice [105]. Moreover, RN486 may also influence the effector function of autoantibodies as implicated by reducing the immune complex-mediated activation of human monocytes in vitro and by downregulating murine macrophage genes induced by interferons in mice [105]. BTK inhibition by ibrutinib significantly decreased B cell and APC activation and reduced the populations of cDCs, macrophages, neutrophils, and MCs, resulting in dampened humoral and cellular autoimmunity in lupus-prone mice [106]. Rankin et al. introduced a novel BTK inhibitor, poseltinib, which effectively prevented lupus-like disease development by inhibiting BCR-mediated signaling and proliferation [107]. A recent study characterized a reversible BTKi, G-744, which modified pathogenic plasma cell signatures and myeloid cell-associated damage in lupus nephritis [108]. A highly selective covalent BTK inhibitor, evobrutinib, has been discovered for the treatment of autoimmune disorders, including SLE [99]. Furthermore, Haselmayer et al. evaluated evobrutinib in preclinical models of SLE and found that it inhibits B cell activation, reduces autoantibody production, and normalizes B and T cell subset constitutions [109]. Clinical studies have investigated the efficacy and safety of BTKis in patients with SLE. A phase II trial of fenebrutinib for SLE demonstrated an acceptable safety profile. However, the primary endpoint, the SLE Responder Index 4 (SRI-4) response, was not met [110]. Another phase II trial of evobrutinib failed to show a treatment effect versus placebo in SLE patients [111]. In China, a phase I/II study of orelabrutinib suggested encouraging efficacy in patients with mild to moderate SLE [112]. Additionally, a phase II trial evaluating the efficacy and safety of upadacitinib (JAK inhibitor) and elsubrutinib alone or in combination in patients with SLE was conducted with the low-dose upadacitinib and elsubrutinib arms ultimately being discontinued due to a lack of efficacy [113]. These studies highlight the ongoing research on BTKis in SLE and the need for further investigation into their differential effectiveness as well as dosing and combination strategies.

#### 4.1.3. Multiple Sclerosis

MS is a central nervous system (CNS) disorder characterized by inflammation, demyelination, gliosis, and neuroaxonal degeneration [114]. While MS has traditionally been understood as primarily driven by T cells, studies revealed that B cells and nearly all types of innate immune cells are also integral in both the disease’s initiation and progression [114]. In mouse models of MS, it has been shown that the BTKi evobrutinib dose-dependently inhibits antigen-triggered activation and maturation of B cells as well as their release of pro-inflammatory cytokines, leading to an impaired capacity of B cells to act as APCs for the development of encephalitogenic T cells, resulting in reduced disease severity [115]. Furthermore, Li et al. reported that BTK inhibition by BIO-0556375 decreases B cell activation and B/T cell interaction via a novel mechanism involving modulation of B cell metabolic pathways, which, in turn, mediates an anti-inflammatory B cell modulation [112]. In fact, a functional link between BTK activity and disease-relevant B cells in patients with MS has been shown [116], which provides further rationale for BTKis to modulate the clinical course of MS. Recently, investigations on the expression of BTK and iron accumulation in myeloid cells in MS brain tissue revealed a consistent correlation of BTK-positive with iron-positive cells across MS lesions [117]. This study also suggested that treatment with evobrutinib not only dampens the associated pro-inflammatory response, but also reduces iron import and storage in activated microglia and macrophages in MS [117].

Of note, Bhargava et al. emphasized the need for appropriate model systems to screen potential therapeutic agents targeting meningeal inflammation in CNS autoimmunity [118]. Utilizing ultra-high field MRI, they demonstrated that evobrutinib reduces meningeal inflammation and leptomeningeal contrast enhancement in a relapsing–remitting experimental model of autoimmune encephalomyelitis [118]. In patients with relapsing MS, evobrutinib at a dose of 75 mg once daily, but not other dosing regimens (75 mg twice daily or 25 mg once daily), reduced the total number of such enhancing MRI lesions [119]. Zurmati et al. conducted a phase IIb trial of tolebrutinib, another brain-penetrant BTKi, in relapsing MS patients, and demonstrated a dose-dependent reduction in new gadolinium-enhancing lesions [120]. Nevertheless, further research is needed to fully understand the mechanisms of action and long-term effects of BTKis in MS therapy.

### 4.2. Infectious Diseases

Given the role of BTK in the differentiation and function of B cells and other immune cells, e.g., macrophages, DCs, neutrophils—which are essential for innate immune responses, including pathogen recognition, phagocytosis, and the production of pro-inflammatory cytokines—BTK inhibition potentially impairs these reactions, leading to increased susceptibility to infections. Indeed, studies have shown that BTK inhibition can impair innate responses against fungal infections in CLL patients [26]. Exposure to ibrutinib and acalabrutinib diminished the signaling pathways activated by *Aspergillus fumigatus*, resulting in an enhanced immunosuppressive signature, deficient phagocytosis, and a significant reduction in the secretion of inflammatory cytokines in macrophages/monocytes from CLL patients over healthy donors [26]. Single-agent treatment with ibrutinib has been associated with an increased risk of atypical *Pneumocystis jirovecii* pneumonia in CLL [121]. Also, in macrophages derived from CLL patients, ibrutinib decreases the secretion of TNF-α and affects polarization, impairing the macrophage-mediated response against *Mycobacterium tuberculosis* [122]. Furthermore, BTK inhibition has been associated with impaired neutrophil effector activity against *Cryptococcus neoformans* and *Aspergillus fumigatus*, considered to increase the risk of invasive fungal infections [123,124]. Notably, the antifungal immunity block imposed by BTK inhibition in neutrophils can be rescued by TNF-α [123]. Moreover, BTK-deficient mononuclear cells from XLA patients showed reduced production of TNF-α in response to lipopolysaccharide, a component of Gram-negative bacteria [125]. In contrast, BTK-deficient macrophages display elevated levels of the pro-inflammatory cytokines TNF-α, IL-6, and IL-12 in *Listeria monocytogenes* infection, suggesting that BTK regulates the extent of the inflammatory response in macrophages in a context-specific manner [126].

Some pathogens cause excessive inflammatory responses in certain cellular compartments, which is a prominent disease-defining effect, with BTK implicated in these processes. For example, significant upregulation of pBTK was observed in human immunodeficiency virus (HIV-1)-infected myeloid cells, and treatment with ibrutinib induced apoptosis in these cells and reduced viral production [127]. Moreover, ibrutinib rescues mice from lethal influenza-induced acute lung injury, highlighting the potential therapeutic implications of targeting BTK in viral infections [128]. Consequently, BTK inhibition in patients with COVID-19 has been a topic of interest. By modulating key transcription factors, BTK may influence macrophage polarization in response to classic M1- and M2-inducing stimuli, potentially mitigating the hyperinflammatory state associated with COVID-19 [129]. Studies have shown that BTKis such as ibrutinib and acalabrutinib may have a protective effect against the pulmonary injury in COVID-19-infected patients [130]. In support, inhibition of BTK in patients with severe COVID-19 has been associated with reduced inflammation and clinical improvement [131]. Additionally, BTKis have been linked to decreased oxygen requirements, decreased hospitalization rates, and shorter durations of hospitalization in COVID-19 patients [132].

Together, the spectrum of BTKis’ effects in infections might be attributed to the different mechanisms of immune responses in various clinical contexts, the specific microorganisms involved, and the different stages of infections.

## 5. Clinical Implications and Future Directions

The immunomodulatory effects of BTKis have significant implications for clinical practice, particularly in the management of CLL, autoimmune diseases, and infections. These inhibitors primarily target B cell signaling pathways, leading to both therapeutic benefits and complex immune system alterations.

In CLL, BTKis provide an effective, targeted therapeutic option with a more favorable toxicity profile than conventional chemo(immuno)therapies [7,133]. Their ability to disrupt BCR signaling results in altered homing alongside reduced proliferation and survival of malignant B cells also involving diminished support by the components of the TME. However, the immunomodulatory effects of these drugs require careful patient management. While they can significantly improve patient outcomes, they also affect immune cell functions, which may increase susceptibility to infections, such as opportunistic infections, as intriguingly shown for ibrutinib in CLL [26,121,122,123]. Importantly, several parameters should be considered when assessing susceptibility in BTKi-treated CLL patients, such as additional individual risk factors (comorbidities), biological features and the extent of the disease, previous/concomitant therapies, and the duration of BTKi exposure. Additionally, the impact of BTKis on other immune cells, such as T cells and macrophages, can potentially lead to unexpected alterations in immune responses, underlining the need for more comprehensive monitoring to advance our understanding of the mechanisms of BTK inhibition in CLL and the importance of a personalized approach to patient management.

In the context of autoimmune diseases, such as RA, SLE, and MS, BTKis present a novel strategy due to their ability to modulate B cell activity and autoantibody production. Recent clinical trials showed promising results for BTKis in MS [119,120]. This may be attributed to the role of B cells in the pathogenesis of MS, particularly in the formation of ectopic lymphoid structures and the production of autoantibodies that contribute to neuroinflammation and demyelination. The ability of BTKis to cross the blood–brain barrier and directly affect CNS inflammation likely adds to their efficacy in MS. While preclinical studies indicated the potential effects of BTKis in SLE and RA, therapeutic benefits have not been observed yet in clinical trials for these conditions.

While BTKis have been shown to also impair innate immune responses mediated by macrophages and neutrophil effector activity, which likely contribute to associated risks of infections, particularly in CLL patients [26,123], they also demonstrated efficacy in reducing infection-associated (hyper)inflammation, e.g., in COVID-19 patients [130,131,132]. Given these ‘dual effects’, it is important to attempt to balance the risks and benefits of BTKi therapy in patients with CLL and other conditions.

Given that BTK inhibition has various effects in different contexts, future research should aim to better understand the mechanisms of BTKis, develop more targeted versions that selectively modulate specific (immune) pathways, clarify the long-term impacts of BTK inhibition on immune function, and explore combination therapies that can better harness the benefits of BTKis. This will be necessary to advance the clinical utility of these small molecules across a broader range of diseases. In fact, as BTK has been shown to regulate activation of the inflammasome, a multiprotein complex involved in innate immune responses [134], it is also a potential molecular target in inflammasome-driven diseases, such as myelodysplastic syndromes (MDSs) [135] or solid tumors (e.g., breast, lung, prostate, colorectal, etc.) [136].

## 6. Conclusions

In this review, we summarized the immunomodulatory mechanisms associated with BTK inhibition, focusing on CLL and other clinical settings. BTK inhibition represents a promising strategy not only for treating B cell malignancies, but also for modulating immune responses. The ongoing exploration of the immunological effects of BTKis will lead to the development of novel therapies that fully utilize the potential of BTK inhibition in the fields of oncology as well as autoimmune and infectious diseases.

## Figures and Tables

**Table 1:** The classes of the selected BTK inhibitors.

Class	Drug Name	Description	Specificity	Toxicity	Approval Status
Covalent	Ibrutinib	First-generation, irreversible at C481	Low	Bleeding, cardiac, hypertension	Approved for CLL, MCL, WM, and MZL
Covalent	Acalabrutinib	Second-generation, irreversible at C481	High	Reduced toxicity vs. ibrutinib	Approved for CLL and MCL
Covalent	Zanubrutinib	Second-generation, irreversible at C481	High	Reduced toxicity vs. ibrutinib	Approved for CLL, MCL, and WM
Covalent	Tirabrutinib	Second-generation, irreversible at C481	High	Insufficient data	Approved in Japan for PCNSL, WM, and LPL
Covalent	Orelabrutinib	Second-generation, irreversible at C481	High	Reduced toxicity vs. ibrutinib	Approved in China for CLL and MCL
Non-covalent	Pirtobrutinib	Reversible non-C481-binding	Very high	Reduced	Approved for R/R MCL
Non-covalent	Nemtabrutinib	Reversible non-C481-binding	Low	Insufficient data	Phase II trials
Non-covalent	Vecabrutinib	Reversible non-C481-binding	Low	Insufficient data	Phase I/II trials
Non-covalent	SHR1459	Reversible non-C481-binding	High	Reduced	Phase II trials in China
Bifunctional	LP-168	Irreversible at wild-type C481, reversible at C481S	Very high	Reduced	Phase II trials
BTK degrader	NX-2127	Protein degrader	Not reported	Insufficient data	Phase I/II trials
BTK degrader	BGB-16673	Protein degrader	Not reported	Insufficient data	Phase I/II trials

Abbreviations: C481 = cysteine 481; C481S = a substitution of cysteine with serine at position 481 of the BTK protein; CLL = chronic lymphocytic leukemia; MCL = mantle cell lymphoma; WM = Waldenström’s macroglobulinemia; MZL = marginal zone lymphoma; R/R = relapsed/refractory; PCNSL = primary central nervous system lymphoma; LPL = lymphoplasmacytic lymphoma.

## Data Availability

Not applicable.

## Supplementary Materials

The following supporting information can be downloaded at https://www.mdpi.com/article/10.3390/cancers16213574/s1, Supplementary Table S1: List of abbreviations used in the manuscript; Supplementary Table S2: Current BTK inhibitors in phase II–III clinical trials for CLL; Supplementary Table S3: BTK inhibitors in clinical trials for systemic lupus erythematosus (SLE), rheumatoid arthritis (RA), and multiple sclerosis (MS).
