# Supplementary material for: The Immunomodulatory Mechanisms of BTK Inhibition in CLL and Beyond
Source: Cancers (Basel). 2024 Oct 23;16(21):3574. doi: 10.3390/cancers16213574 (PMC11545099; doi:10.3390/cancers16213574)
Supplement: Supplementary file 1 [file cancers-16-03574-s001.zip › cancers-3264099-supplementary.pdf]

Supplemental Table S1. List of abbreviations used in the manuscript.

Abbreviation	Full Name
**AKT**	Protein kinase B
**APC**	Antigen-presenting cell
**BCR**	B-cell receptor
**BTK**	Bruton's tyrosine kinase
**BTKi's**	Bruton's tyrosine kinase inhibitors
**C481**	Cysteine 481
C481S	A substitution of cysteine with serine at position 481 of the BTK protein
**CAM**	Calmodulin
**CAR**	Chimeric antigen receptor
**COVID-19**	Coronavirus disease 2019
**CLL**	Chronic lymphocytic leukemia
**CNS**	Central nervous system
**DAG**	Diacylglycerol
**DC**	Dendritic cell
**EGFR**	Epidermal growth factor receptor
**ERK**	Extracellular signal-regulated kinase
**FcγR**	Fc gamma receptor
**FcεRI**	High-affinity IgE receptor
**GM-CSF**	Granulocyte-macrophage colony-stimulating factor receptor
**GvHD**	Graft-versus-host disease
**HIV-1**	Human immunodeficiency virus
**HLH**	Hemophagocytic lymphohistiocytosis
**IL-6**	Interleukin-6
**LPS**	Lipopolysaccharide
**IP3**	Inositol 1,4,5-trisphosphate
**ITK**	Interleukin-2-inducible T-cell kinase
**ITAM**	Immuno-receptor tyrosine-based activation motif
**LYN**	Lyn kinase
**MAPK**	Mitogen-activated protein kinase
**MC**	Mast cell
**MCL**	Mantle cell lymphoma
**M-CSF**	Macrophage colony-stimulating factor
**MDS**	Myelodysplastic syndromes
**MDSC**	Myeloid-derived suppressor cells
**MHC**	Major histocompatibility complex
**MS**	Multiple sclerosis
**MSC**	Mesenchymal stromal cells
**mTOR**	Mechanistic target of rapamycin
**MYD88**	Myeloid differentiation primary response 88
**MZL**	Marginal zone lymphoma
**M1**	Pro-inflammatory macrophage
**M2**	Anti-inflammatory macrophage
**NFAT**	Nuclear factor of activated T cells
**NFκB**	Nuclear factor kappa-light-chain-enhancer of activated B cells
**NLC**	Nurse-like cell
Notch-c-Myc-EZH2	Notch signaling pathway - Myelocytomatosis oncogene - Enhancer of zeste homolog 2
**PCNSL**	Primary central nervous system lymphoma
**PD-1**	Programmed cell death protein 1
**PD-L1**	Programmed cell death ligand 1
**PI3K**	Phosphoinositide 3-kinase
**PLCγ2**	Phospholipase C gamma 2
**PKC**	Protein kinase C
**RA**	Rheumatoid arthritis
**R/R**	Relapsed/refractory
**SHIP1**	SH2-containing inositol-5′-phosphatase 1
**SLE**	Systemic lupus erythematosus
**STAT3**	Signal transducer and activator of transcription 3
**SYK**	Spleen tyrosine kinase
**SRI-4**	SLE Responder Index 4

**TAM**	Tumor-associated macrophages
**TCL1A**	T-cell leukemia/lymphoma 1A
**TEC**	Tyrosine kinase expressed in hepatocellular carcinoma
**Th**	T helper cells
**TLR**	Toll-like receptor
**TME**	Tumor microenvironment
**TNF-α**	Tumor necrosis factor alpha
**Treg**	Regulatory T cells
**VLA-4**	Very late activation antigen 4
**VCAM-1**	Vascular cell adhesion molecule 1
**WM**	Waldenström's macroglobulinemia
**XLA**	X-linked agammaglobulinemia

Supplemental Table S2. Current BTK inhibitors in phase II-III clinical trials for CLL. The table summarizes the current BTK inhibitors (including ibrutinib, acalabrutinib, zanubrutinib, and pirtobrutinib) that are under phase II-III clinical trials for the treatment of CLL. These trials explore BTK inhibitors as monotherapies and in combination with agents like venetoclax, obinutuzumab, rituximab, and chemotherapy, in various CLL patient cohorts, including untreated and relapsed/refractory cases, those with resistance mutations, measurable residual disease, high-risk cytogenetic profiles such as del(17p), and elderly patients (age ⩾65 years). These studies aim to evaluate the efficacy and safety of BTK inhibitors, and their potential synergistic effects, to improve treatment outcomes for CLL patients.

Treatment	Target Patient Population	Phases	NCT Number
**Ibrutinib**			
Ibrutinib / Obinutuzumab + Venetoclax	untreated	II	NCT05105841
Ibrutinib + Venetoclax	R/R	II	NCT04754035
Ibrutinib + Venetoclax induction, followed by Ibrutinib + Obinutuzumab	patients with MRD	II	NCT04639362
Ibrutinib monotherapy vs Ibrutinib + Venetoclax vs Venetoclax + Obinutuzumab	untreated	III	NCT04608318
Ibrutinib + Daratumumab	R/R	II	NCT04230304
Ibrutinib + Venetoclax	untreated intermediate-risk CLL	II	NCT04010968
Ibrutinib + Venetoclax + Obinutuzumab	untreated, age >= 65 years	III	NCT03737981
Ibrutinib + Daratumumab	R/R	II	NCT03734198
Ibrutinib lead-in followed by Ibrutinib + Venetoclax	R/R	II	NCT03708003
Ibrutinib + Venetoclax + Obinutuzumab vs Ibrutinib + Obinutuzumab	untreated	III	NCT03701282
Ibrutinib + Venetoclax	CLL with resistance mutations	II	NCT03513562
Ibrutinib + Venetoclax vs Obinutuzumab + Chlorambucil	untreated	III	NCT03462719
Ibrutinib + Venetoclax	R/R	II	NCT03226301
Ibrutinib + Fludarabine and Pembrolizumab	R/R or high risk	II	NCT03204188
Ibrutinib + Cirmtuzumab	treated/untreated	I/II	NCT03088878
Ibrutinib + Venetoclax	R/R and untreated	II	NCT02756897
Ibrutinib monotherapy	treated	II	NCT02649387
Ibrutinib + Obinutuzumab + Fludarabine phosphate + Cyclophosphamide	treated	II	NCT02629809
Ibrutinib + vaccine therapies	untreated	II	NCT02518555
Ibrutinib + short-course Fludarabine	untreated	II	NCT02514083
Ibrutinib + Obinutuzumab + GDC-0199	R/R and untreated	1/2	NCT02427451
Ibrutinib + Ofatumumab	treated	2	NCT02388048
Ibrutinib + Obinutuzumab	untreated	I/II	NCT02315768
Ibrutinib + Fludarabine + Cyclophosphamide + Rituximab	untreated	II	NCT02251548
Ibrutinib + Rituximab	untreated	II	NCT02232386
Ibrutinib + Rituximab vs Fludarabine Phosphate + Cyclophosphamide + Rituximab	untreated	III	NCT02048813
Ibrutinib +/- Rituximab	R/R	II	NCT02007044
Ibrutinib alone vs Ibrutinib + Rituximab vs Bendamustine + Rituximab	untreated or age ≥65 years	III	NCT01886872
Monotherapy	R/R	II	NCT01589302
Monotherapy	age ≥65 years or with del17p	II	NCT01500733
**Acalabrutinib**			
Acalabrutinib + Venetoclax vs Obinutuzumab + Venetoclax	untreated	III	NCT05057494
Monotherapy	age ≥80 years	II	NCT04883749
Acalabrutinib + Obinutuzumab	untreated/treated	II	NCT04722172
Acalabrutinib before alloSCT	R/R	II	NCT04716075

Monotherapy	CLL patients with R/R AIHA	II	NCT04657094
Acalabrutinib + Umbralisib + Ublituximab	relapsed and untreated	II	NCT04624633
Monotherapy	early stage CLL with high risk	III	NCT04178798
Acalabrutinib vs Chlorambucil + Rituximab	untreated	III	NCT04075292
Monotherapy	R/R and untreated	III	NCT04008706
Monotherapy	R/R	I/II	NCT03932331
Acalabrutinib + Venetoclax vs Acalabrutinib + Venetoclax +/- Obinutuzumab vs chemoimmunotherapy	untreated	III	NCT03836261
Acalabrutinib + high-frequency low-dose subcutaneous Rituximab	untreated	II	NCT03788291
Acalabrutinib + Venetoclax + Obinutuzumab	untreated	II	NCT03580928
Acalabrutinib vs Idelalisib + Rituximab vs Bendamustine + Rituximab	R/R	III	NCT02970318
Acalabrutinib vs Ibrutinib	R/R	II	NCT02717611
Acalabrutinib vs Ibrutinib	treated	III	NCT02477696
Acalabrutinib +/- Obinutuzumab vs Obinutuzumab + Chlorambucil	untreated	III	NCT02475681
Acalabrutinib + Pembrolizumab	treated/untreated	II	NCT02362035
Monotherapy	R/R and untreated del17p CLL	II	NCT02337829
Acalabrutinib + ACP-31	treated/untreated	I/II	NCT02328014
Monotherapy	R/R	I/II	NCT02029443
**Zanubrutinib**			
Zanubrutinib + Venetoclax + Obinutuzumab following Bendamustine	R/R	II	NCT04515238
Zanubrutinib + BGB-10188	R/R	I/II	NCT04282018
Monotherapy	R/R	I/II	NCT04172246
Zanubrutinib vs Bendamustine + Rituximab	untreated	III	NCT03336333
**Pirtobrutinib**			
Pirtobrutinib vs Ibrutinib	untreated/treated	III	NCT05254743
Pirtobrutinib vs Bendamustine + Rituximab	untreated	III	NCT05023980
Monotherapy	treated	II	NCT04849416
Pirtobrutinib vs Idelalisib + Bendamustine or Rituximab + Rituximab	treated	III	NCT04666038
Monotherapy	treated	I/II	NCT03740529

Abbreviations: R/R, relapse/refractory; MRD, minimal residual disease; AIHA, autoimmune hemolytic anemia; del, deletion.

Supplemental Table S3. BTK inhibitors in clinical trials for systemic lupus erythematosus (SLE), rheumatoid arthritis (RA), and multiple sclerosis (MS). The table presents an overview of clinical trials investigating BTK inhibitors, including Fenebrutinib, Evobrutinib, Orelabrutinib, Elsubrutinib, BMS-986142, and Tolebrutinib, for the treatment of RA, SLE, and MS, including both ongoing and completed studies.

**Treatment and Target Patient Population**	**Study Status**	**Phases**	**NCT Number**
**Fenebrutinib**			
Relapsing MS	Active	II	NCT05119569
Relapsing MS	Active	III	NCT04586010
Relapsing MS	Active	III	NCT04586023
Primary progressive MS	Active	III	NCT04544449
SLE	Completed	II	NCT02908100
**Evobrutinib**			
Relapsing MS	Terminated	III	NCT04338022
Relapsing MS	Terminated	III	NCT04338061
R-R MS	Terminated	III	NCT04032158
R-R MS	Terminated	III	NCT04032171
RA	Completed	II	NCT03233230
R-R MS	Terminated	II	NCT02975349
SLE	Terminated	II	NCT02975336
RA	Completed	II	NCT02784106
SLE	Completed	I	NCT02537028
**Orelabrutinib**			
SLE	Active	II	NCT05688696
R-R MS	Active	II	NCT04711148
SLE	Completed	I/II	NCT04305197
**Elsubrutinib**			
SLE	Completed	II	NCT04451772
SLE	Completed	II	NCT03978520
RA	Terminated	II	NCT03823378
RA	Completed	II	NCT03682705
**BMS-986142**			
RA	Completed	I	NCT02762123
RA	Completed	II	NCT02638948
RA	Completed	I	NCT02456844
**Tolebrutinib**			
Relapsing MS / Secondary progressive MS / Progressive relapsing	Active	III	NCT06372145
MS	Completed	I	NCT06106074
MS	Completed	I	NCT06064539
MS	Active	II	NCT04742400
Primary progressive MS	Active	III	NCT04458051
Secondary progressive MS	Active	III	NCT04411641
Relapsing MS	Active	III	NCT04410991
Relapsing MS	Active	III	NCT04410978
Relapsing MS	Active	II	NCT03996291

Abbreviations: R-R, relapsing-remitting.
